# Adductor Canal Block for Postoperative Pain Treatment after Revision Knee Arthroplasty: A Blinded, Randomized, Placebo-Controlled Study

**DOI:** 10.1371/journal.pone.0111951

**Published:** 2014-11-11

**Authors:** Pia Jæger, Zbigniew J. Koscielniak-Nielsen, Henrik M. Schrøder, Ole Mathiesen, Maria H. Henningsen, Jørgen Lund, Morten T. Jenstrup, Jørgen B. Dahl

**Affiliations:** 1 Department of Anaesthesia, Centre of Head and Orthopaedics, Copenhagen University Hospital, Rigshospitalet, Copenhagen, Denmark; 2 Department of Orthopaedic Surgery, Centre of Head and Orthopaedics, Copenhagen University Hospital, Rigshospitalet, Copenhagen, Denmark; 3 Section of Acute Pain Management, Department of Anaesthesia, Centre of Head and Orthopaedics, Copenhagen University Hospital, Rigshospitalet, Copenhagen, Denmark; 4 Department of Anaesthesia, Aleris-Hamlet Hospitals, Copenhagen, Denmark; Harvard Medical School, United States of America

## Abstract

**Background:**

Revision knee arthroplasty is assumed to be even more painful than primary knee arthroplasty and predominantly performed in chronic pain patients, which challenges postoperative pain treatment. We hypothesized that the adductor canal block, effective for pain relief after primary total knee arthroplasty, may reduce pain during knee flexion (primary endpoint: at 4 h) compared with placebo after revision total knee arthroplasty. Secondary endpoints were pain at rest, morphine consumption and morphine-related side effects.

**Methods:**

We included patients scheduled for revision knee arthroplasty in general anesthesia into this blinded, placebo-controlled, randomized trial. Patients were allocated to an adductor canal block via a catheter with either ropivacaine or placebo; bolus of 0.75% ropivacaine/saline, followed by infusion of 0.2% ropivacaine/saline. Clinicaltrials.gov ID: NCT01191593.

**Results:**

We enrolled 36 patients, of which 30 were analyzed. Mean pain scores during knee flexion at 4 h (primary endpoint) were: 52±22 versus 71±25 mm (mean difference 19, 95% CI: 1 to 37, P = 0.04), ropivacaine and placebo group respectively. When calculated as area under the curve (1–8 h/7 h) pain scores were 55±21 versus 69±21 mm during knee flexion (P = 0.11) and 39±18 versus 45±23 mm at rest (P = 0.43), ropivacaine and placebo group respectively. Groups were similar regarding morphine consumption and morphine-related side effects (P>0.05).

**Conclusions:**

The only statistically significant difference found between groups was in the primary endpoint: pain during knee flexion at 4 h. However, due to a larger than anticipated dropout rate and heterogeneous study population, the study was underpowered.

**Trial Registration:**

Clinicaltrials.gov NCT01191593

## Introduction

Revision total knee arthroplasty (TKA) is a challenge, both for surgeons and anesthesiologists. For the surgeon the procedure is technically more difficult, takes longer time, and has a higher rate of complications than primary TKA [1]. For the anesthesiologist the main challenge lies in handling the postoperative pain treatment. Compared with primary TKA, revision TKA is assumed to be even more painful. Many patients suffer from intense preoperative pain [2], often treated with opioids, adding to the complexity of the task. With an increasing volume of primary TKAs being performed, the number of revisions is also inevitably rising; nonetheless, to our knowledge no pain studies have been performed in this subgroup of patients.

The femoral nerve block has been the mainstay for postoperative pain treatment following knee replacement for decades. Current trends however, focus on providing analgesia with minimal motor block. Contrary to the femoral nerve block, the adductor canal block (ACB) is predominantly a sensory nerve block, preserving quadriceps muscle strength and mobilization ability [3]–[4]. In patients undergoing primary TKA, the ACB reduced postoperative pain and morphine consumption, and enhanced ambulation compared with placebo [5]–[8].

We hypothesized that the adductor canal block would also reduce postoperative pain following revision TKA. Hence the primary aim of this study was to examine the effect of adductor canal block on pain during knee flexion after revision TKA compared with placebo. Secondary endpoints were pain at rest, morphine consumption and morphine-related side effects.

## Methods

### Ethics statement

This prospective, randomized, blinded, placebo-controlled trial was approved by the local Regional Ethics Committee (H-3-2010-063), the Danish Medicines Agency (2010-021161-71), the Danish Data Protection Agency and registered at clinicaltrials.gov (NCT01191593). After obtaining written informed consent, we recruited patients scheduled for revision TKA from August 2010–March 2013. The study was conducted at the Centre of Head and Orthopaedics, Copenhagen University Hospital, Rigshospitalet, Copenhagen, Denmark and monitored by the Copenhagen University Hospital GCP (Good Clinical Practice) unit. Data are presented in accordance with the CONSORT (Consolidated Standards of Reporting Trials) statement. The protocol for this trial and supporting CONSORT checklist are available as supporting information; see Checklist S1 and Protocol S1.

Eligible patients were Danish speaking adults, scheduled for revision TKA in general anesthesia, aged 40–85 years, with American Society of Anesthesiologists physical status I–III, and body mass index of 18–40 kg/m^2^. Exclusion criteria were inability to cooperate, allergy to any drug used in the study, alcohol or drug abuse.

### Interventions

Premedication, consisting of 1 g acetaminophen, was given orally one hour before surgery. General anesthesia was induced with propofol, and maintained with propofol (variable rate) and remifentanil 30 µg/kg/h (fixed rate). Thirty minutes prior to the end of surgery, patients were given morphine 0.2 mg/kg intravenously. A femoral tourniquet was applied peri-operatively and intraoperative fluids were administered at the discretion of the anesthetist.

The adductor canal block was performed after the end of surgery, while maintaining general anesthesia until completion of block procedure. A 12L-SC linear ultrasound transducer (GE Medical Systems, Wuxi, China) was placed on the medial part of the thigh, halfway between the superior anterior iliac spine and the patella with the leg slightly externally rotated. The femoral artery was identified in short axis in the adductor canal, underneath the sartorius muscle. After skin disinfection with chlorhexidine gluconate and isopropyl alcohol an 18-gauge, Contiplex Tuohy needle (B.Braun Medical, Melsungen, Germany) was inserted in-plane from the lateral side of the transducer. The needle tip was placed underneath the sartorius muscle, just lateral to the artery and saphenous nerve, using 2–3 ml of saline to ensure correct placement (directional terms are referenced from the US image). A 20-gauge catheter was then advanced 2 cm beyond the tip of the needle, and 30 ml of study medication was slowly injected via the catheter, with repeated aspiration. The position of the catheter tip was adjusted during injection to obtain a semicircular expansion between the sartorius fascia and the artery.

In a double masked fashion patients were allocated to ACB with ropivacaine or placebo (saline) for 24 hours. We administered study medication as an initial bolus of 30 ml 0.75% ropivacaine or saline, followed by another bolus of 15 ml 6 hours later. Immediately after the second bolus, we started infusion of study medication (0.2% ropivacaine or saline) at a rate of 8 ml/h.

Postoperatively, all patients received oral acetaminophen (1 g) and ibuprofen (400 mg) every six hours and a patient-controlled analgesia (PCA) pump with morphine intravenously (bolus 2.5 mg, lock-out time 10 minutes, no background infusion). If analgesia was inadequate patients received an additional bolus of 2.5 mg morphine and/or 0.05 mg fentanyl intravenously until adequate analgesia was obtained (0.1 mg of fentanyl was considered equipotent with 10 mg of morphine). Patients with a daily intake of strong opioids (morphine, oxycodone, methadone, fentanyl, ketobemidone) received their habitual analgesics during the study period. These doses were not included in the cumulative morphine consumption. In case of moderate to severe postoperative nausea or vomiting (PONV), patients received 4 mg of ondansetron intravenously, with supplemental doses of 1 mg, when needed.

### Outcomes

The primary endpoint was pain during 45 degrees flexion of the knee at 4 hours postoperatively.

Secondary endpoints were pain at rest and during flexion of the knee (calculated as area under the curve, 1–8 h and at 24 h), cumulative morphine consumption (0–24 h, 0–8 h and 8–24 h), number of vomiting episodes, ondansetron consumption and degree of nausea and sedation (mean value 1–8 h and 24 h).

### Assessment of outcomes

We assessed all outcomes at 1, 2, 4, 6, 8 and 24 h postoperatively. Pain intensity was assessed on a visual analogue scale (VAS) with 0 mm  =  no pain, and 100 mm  =  worst imaginable pain. Patients rated nausea and sedation on a four-point scale (0 =  no nausea/sedation, 1 =  light, 2 =  moderate, 3 =  severe), and reported the number of vomiting episodes (volume greater than 10 ml).

To counter the problem of tolerance in patients treated with opioids, we decided that patients with a daily intake of strong opioids should continue with their habitual analgesics during the study period, and that these doses should not be included in the cumulative morphine consumption.

### Randomization and blinding

The pharmacy prepared the study medication in identical pre-packed boxes, consecutively numbered according to a computer generated block randomization list (1∶1 ratio, blocks of 10). Subjects were assigned consecutive numbers upon inclusion to the study and received the study medication from the corresponding boxes. A research fellow neither involved in the study nor in the care of the patient administered the study medication in unlabeled syringes for injection and unmarked drug bags for infusion, before handing it over to the investigators. Ropivacaine and saline are identical in appearance.

All investigators, staff and patients were blinded to the treatment groups. The randomization key was first broken after all patients were enrolled, data computed and statistical analyses performed.

### Statistics

We considered a difference of 20 mm in VAS pain scores to be clinically relevant. Assuming a SD of 20, with α = 0.05 and power  = 0.80; 17 patients in each group were required to detect a difference of 20 mm in VAS pain scores. We planned for an inclusion of 36 subjects to compensate for dropouts.

Statistical analyses were conducted using SPSS 19 (SPSS, Chicago, Illinois, USA). Continuous variables are presented as mean ±SD or with medians and 10th–90th percentiles, as appropriate, and ordinal and nominal variables as n (%). The Kolmogorov-Smirnov test was used to assess whether data were normally distributed. If the assumption of normality was met, comparisons between the groups were performed using independent samples t-test; if the assumption of normality was rejected, comparisons were made using Mann-Whitney U test for unpaired data. Nausea and sedation scale scores were compared at 24 h, and by using the mean score from each patient from 1–8 h. We calculated the area under the curve (AUC) for the interval 1–8 h, for VAS pain scores during knee flexion and at rest. Categorical data (ondansetron consumption and vomiting episodes, 0–24 h) were analyzed using the Chi-squared test or by Fischer exact test, as appropriate. The nature of the hypothesis testing was two-tailed, and P<0.05 was considered statistically significant.

### Post hoc analysis

We performed a linear mixed model including pain scores at 1, 2, 4, 6 and 8 hours (originally analyzed as AUC), where we included time as a repeated effect with a first order autoregressive covariance structure (AR(1)). Treatment, time and the interaction treatment*time were set as fixed effects, and preoperative pain scores were included as covariates to compensate for baseline differences. This analysis was performed as an intention to treat analysis.

## Results

**Figure 1:**
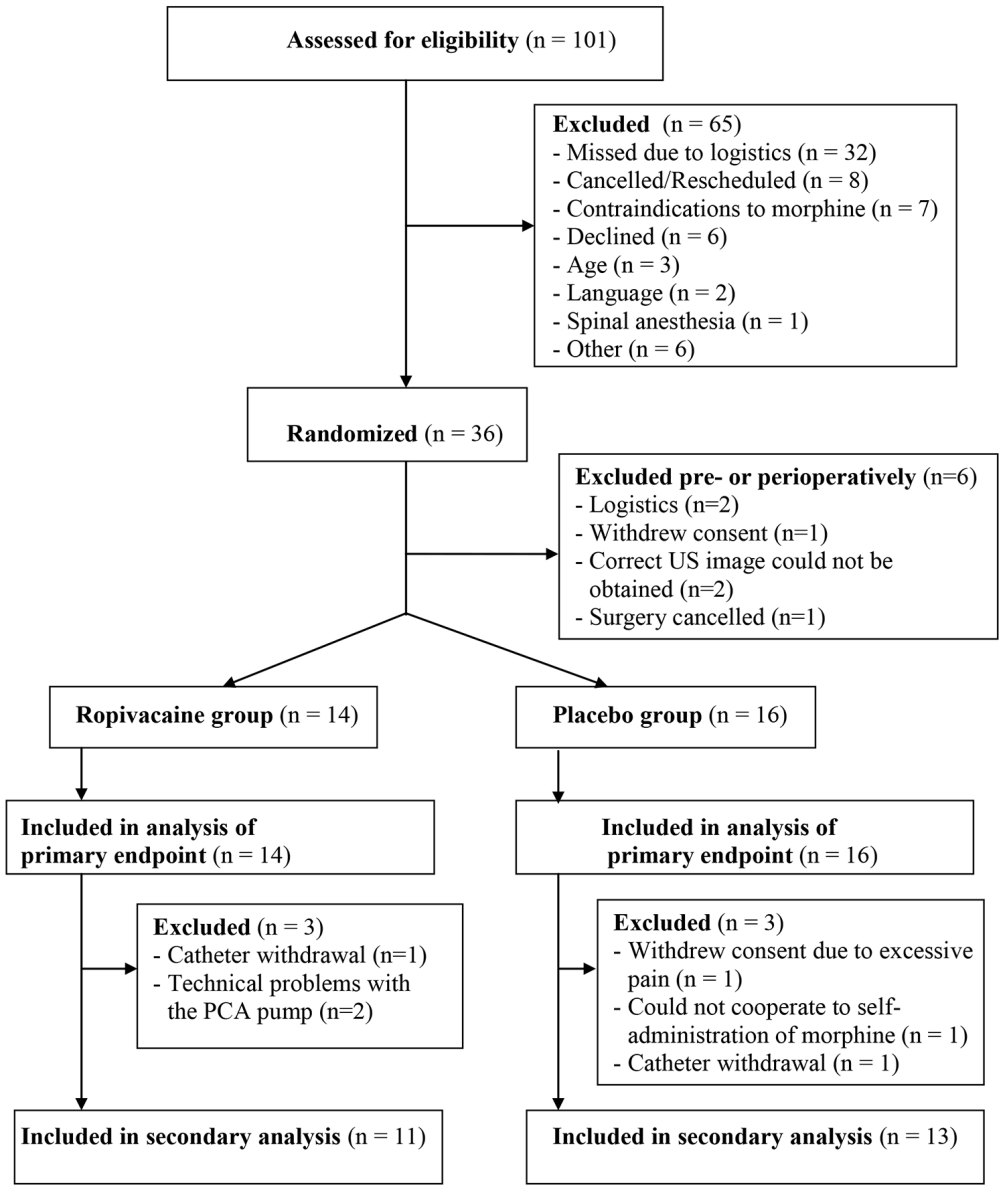
Flow diagram of patient distribution. US =  ultra sound. PCA =  patient-controlled analgesia.

We enrolled and randomized 36 patients during a 31-month period, beginning August 2010; 6 patients were excluded prior to receiving any treatment, 2 patients were excluded between 4 and 6 h, and another 4 patients were excluded after completion of the first 8 h of the study protocol. This resulted in 30 patients with data for our primary endpoint (intention to treat analysis): VAS during flexion of the knee at 4 h postoperatively. As for the secondary outcomes all available data were analyzed, although missing data for the final end point in cumulative morphine consumption and pain scores calculated as AUC will in practice render these analyses per protocol analyses. The details of patient flow through the study, including details on patient exclusions, are presented in Figure 1 (CONSORT flow chart). Patients' demographics and perioperative data are presented in Table 1 and 2.

**Table 1:** Patient characteristics.

	Ropivacaine group	Placebo group
**Number of patients**	14	16
**Sex (male/female)**	8/6	8/8
**Age (years)**	65 (50–78)	67 (42–83)
**Height (cm)**	170 (162–184)	170 (150–191)
**Weight (kg)**	94 (69–111)	88 (58–115)
**Preoperative VAS** * **pain score at rest**		
VAS score in mm	9 (0–67)	17 (0–75)
*VAS score in proportions:*		
VAS<30 mm	6/14 (43)	7/15 (47)
VAS 30–59 mm	4/14 (29)	5/15 (33)
VAS>60 mm	4/14 (29)	3/15 (20)
**Preoperative VAS** * **pain score at 45 degrees knee flexion**		
VAS score in mm	44 (5–76)	30 (0–78)
*VAS score in proportions:*		
VAS<30 mm	10/14 (71.5)	11/16 (69)
VAS 30–59 mm	3/14 (21.5)	3/16 (19)
VAS>60 mm	1/14 (7)	2/16 (12)
**Habitual analgesics:**		
None	0/14 (0)	3/16 (19)
Paracetamol and/or ibuprofen	5/14 (36)	1/16 (6)
Weak opioids†	4/14 (28)	10/16 (62.5)
Daily intake of strong opioids‡	5/14 (36)	2/16 (12.5)

Values are reported as number of subjects, proportions (percentage) or median (10–90%).

*VAS =  visual analogue scale.

† Weak opioids  =  codeine or tramadol, or a non-daily intake of strong opioids.

‡ Strong opioids  =  morphine, oxycodone, methadone, fentanyl, ketobemidone.

**Table 2:** Perioperative data.

	Ropivacaine group	Placebo group
**Number of patients**	14	16
**Operated side (right/left)**	8/6	6/10
**Duration of surgery (min)**	146 (82–194)	138 (100–164)
**Order of revision performed:**		
1. Revision	6/14 (43)	8/16 (50)
2. Revision	5/14 (36)	6/16 (38)
3. Revision	3/14 (21)	1/16 (6)
4. Revision	0/14 (0)	1/16 (6)
**Reason for revision TKA** * **:**		
Septic loosening	2/14 (14)	4/14 (25)
Aseptic loosening	6/14 (43)	5/14 (31)
Component malposition	3/14 (21.5)	2/14 (12.5)
Instability	3/14 (21.5)	3/14 (19)
Stiffness	0/14 (0)	2/14 (12.5)
**Blood loss (ml)**	150 (0–450)	200 (0–480)
**Isotonic sodium chloride (ml)**	1250 (650–2625)	1250 (520–2010)
**Hydroxyethyl starch colloids (ml)**	0 (0–350)	0 (0–500)

Values are reported as number of subjects, proportions (percentage) or median (10–90%).

*TKA =  total knee arthroplasty.

**Figure 2:**
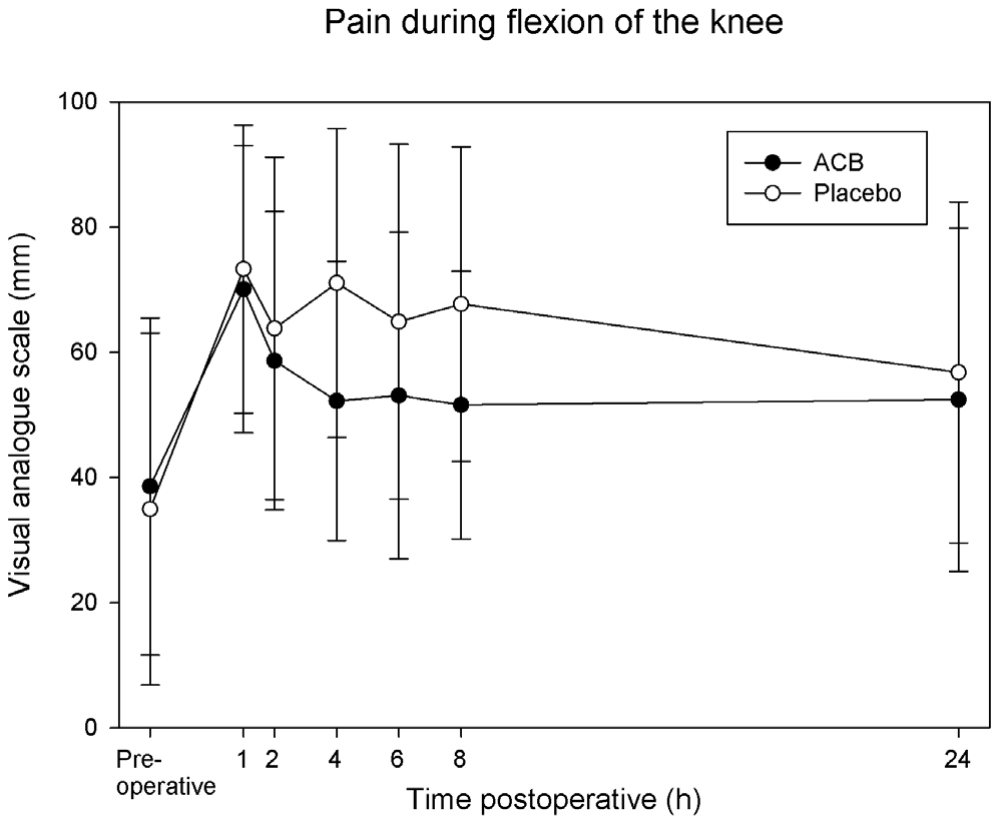
Effects of the adductor canal block on pain during 45 degrees flexion of the knee. Pain was assessed with a visual analogue scale (0–100 mm; with 0 equal no pain and 100 being the worst imaginable pain). Pain scores were lower in the ropivacaine group at 4 hours (primary endpoint (P = 0.04), but not when calculated as area under the curve (1–8 h, P = 0.11). Data are expressed as mean ±SD. ACB =  adductor canal block.

**Figure 3:**
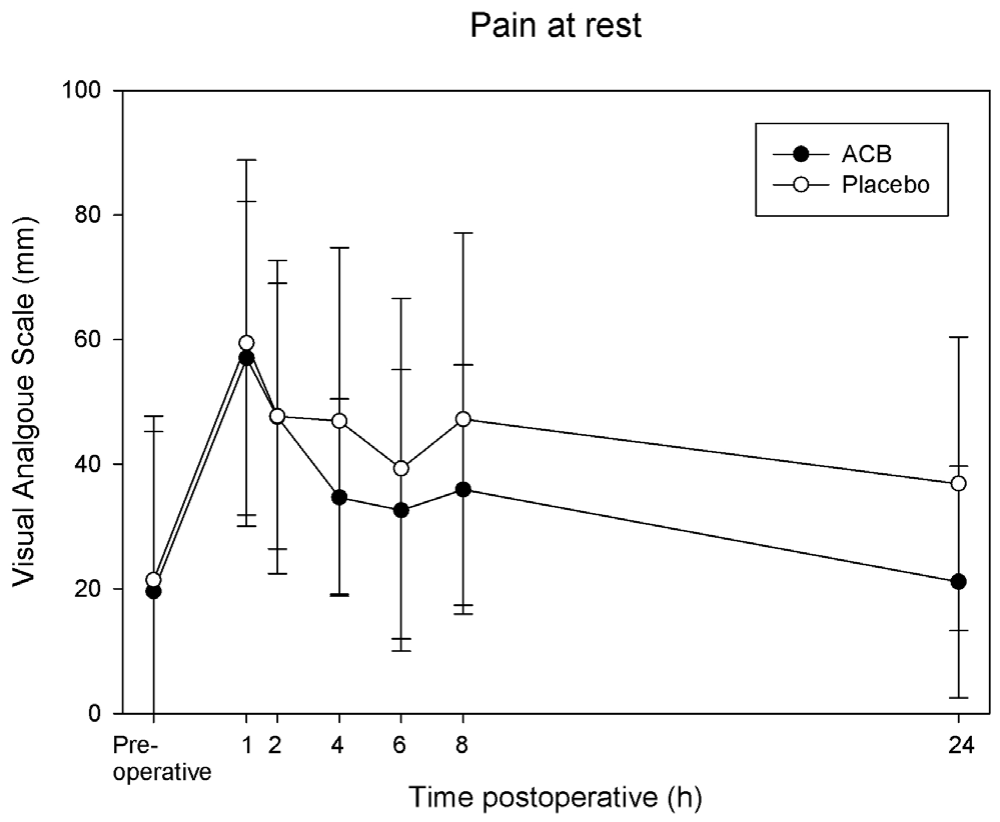
Effects of the adductor canal block on pain at rest. Pain was assessed with a visual analogue scale (0–100 mm; with 0 equal no pain and 100 being the worst imaginable pain). Comparisons between the groups were made as area under the curve (AUC) for the interval 1–8 h postoperatively, showing no statistically significant difference between the groups (P = 0.43). Data are expressed as mean ±SD. ACB =  adductor canal block.

VAS pain scores during flexion of the knee at 4 h postoperatively (primary endpoint) were 52±22 mm in the ropivacaine group and 71±25 mm in the placebo group (mean difference 19, 95% CI: 1 to 37, P = 0.04). At 24 h, pain scores were 50±27 vs. 57±27 mm during knee flexion (mean difference 7, 95% CI: −16 to 30, P = 0.53) and 21±19 vs. 37±24 mm at rest (mean difference 16, 95% CI: −2 to 34, P = 0.09) at rest; ropivacaine and placebo group respectively. When calculated as AUC for the interval 1–8 h/7 h, VAS pain scores during flexion of the knee were 55±21 mm in the ropivacaine group compared with 69±21 mm in the placebo group (mean difference 14, 95% CI: −3 to 31, P = 0.11, Figure 2). At rest, mean pain scores (AUC for the interval 1–8 h/7 h) were 39±18 mm in the ropivacaine group compared with 45±23 mm in the placebo group (mean difference 6, 95% CI: −9 to 22, P = 0.43, figure 3).

Cumulative total morphine consumption was 61±38 versus 74±48 mg from 0–24 h (P = 0.48), 43±34 vs. 44±28 mg at 0–8 h (P = 0.97) and 28±30 vs. 29±18 mg from 8–24 h (P = 0.95), ropivacaine and placebo group respectively (Table 3). There were no differences between the groups regarding morphine-related side effects: nausea (1–8 h: 0.15 vs. 0.24, P = 0.33; 24 h: 0.18 vs. 0.46, P = 0.44), sedation (1–8 h: 1.1 vs. 1.1, P = 0.83; 24 h: 0.64 vs. 0.85, P = 0.50), vomiting episodes (6/16 vs. 6/14 P = 0.77), or in the need of antiemetics (8/16 vs. 7/14, P = 0.46), placebo and ropivacaine group respectively.

**Table 3:** Morphine consumption.

Cumulative total morphine consumption (mg):	Ropivacaine group	Placebo group
0–1 h postoperative	5 (0–40)	5 (0–39)
0–2 h postoperative	19 (3–64)	25 (5–51)
0–4 h postoperative	28 (5–91)	34 (5–61)
0–6 h postoperative	35 (7–105)	35 (4–73)
0–8 h postoperative	35 (7–114)	39 (4–89)
0–24 h postoperative	55 (8–135)	60 (6–142)

Values are presented as medians (10–90 percentiles).

All data used for analysis are available in the file: Data S1.

There were no adverse events in the study population.

### Post hoc analysis

Daily intake of strong opioids was omitted from total morphine consumption to counter the problem of tolerance. By chance, more patients in the ropivacaine group had a daily intake of strong opioids (36% vs. 13%, Table 1). If we had included the habitual analgesics from these patients, the cumulative total morphine consumption (0–24 h) would have been 64±39 mg in the ropivacaine group and 81±57 mg in the placebo group (P = 0.43).

Linear mixed models analyses (based on intention to treat) showed a tendency of lower pain scores in the ropivacaine group during knee flexion for the repeated measurements at 1, 2, 4, 6 and 8 hours (estimated difference of 19 mm, P = 0.06) but not for pain at rest (estimated difference 11 mm, P = 0.35).

## Discussion

The ACB reduces pain scores and morphine consumption compared with placebo after primary TKA [5]–[8]. In this study of ACB for revision TKA, the only statistically significant difference was seen in our primary endpoint: pain during flexion of the knee at 4 h postoperatively. At this time point there was a 19 mm difference in pain scores between the groups. This is very close to what we *a priori* considered to be clinically relevant (20 mm), and what can be expected in a multimodal analgesic setting with patients receiving acetaminophen, ibuprofen and morphine. Pain scores were consistently lower in the ropivacaine group, but mean differences varied between 6–19 mm.

To our knowledge, this is the first postoperative pain study performed exclusively in patients undergoing revision knee arthroplasty. In most studies revision TKA is an exclusion criteria, probably because this group of patients are assumed to have a more complex pathology, and more complex pain problems. As the number of revision TKAs is steadily increasing, we believe it is necessary to perform specific studies in this subgroup of patients.

A major limitation of this study is a larger dropout rate than anticipated, resulting in a reduced study size, especially affecting the secondary outcomes. Six patients were excluded before receiving an ACB. Because allocation assignment was blinded to all investigators and patients, and these six patients neither received an ACB nor any study medication, their exclusion was due to chance and is not likely to have biased our results. Prolonging the study period, although desirable, was not feasible because we stopped performing revision TKAs at our hospital. For our primary endpoint this resulted in a study population four patients short of our *a priori* calculated sample size of n = 17 in each group. Although the exclusion of these first six patients is not likely to have biased our results, additional six patients were excluded after obtaining our primary endpoint at 4 h postoperatively (for further details please see Figure 1), resulting in complete datasets for only 24 subjects for the secondary analysis. This secondary exclusion may have biased the results of our secondary endpoints, as these data points may not be missing at random. Especially the two patients in the placebo group, excluded due to excessive pain and difficulty in self-administration of morphine, may have biased our results. Nevertheless, these patients were included in the post-hoc analyses using the linear mixed model.

Both total morphine consumption and calculation of AUC requires that the final observation is not missing, because extrapolation of morphine consumption or imputation of data by method of last observation carried forward has been widely criticized [9]. Unfortunately, these limitations will in practice render such analyses per protocol analyses. One of the major advantages of mixed models is that it does not require full data sets, thus allowing an intention to treat analysis. To compensate for the relatively large portion of missing data for our secondary analysis and the large variability in preoperative VAS pain scores, we performed a linear mixed model analysis re-analyzing the repeated VAS pain scores from 1–8 hours (originally compared by calculating the AUC). This revealed a tendency towards lower pain scores during knee flexion in the ropivacaine group (estimated difference 19 mm, P = 0.06), but not for pain at rest (estimated difference 11, P = 0.35).

Postoperative pain treatment following revision TKA is assumed to be more complex than after primary TKA. The patients suffer more complications, have extended hospital stays, and they are assumed to be in more pain [1]. A previous study, reports of mean preoperative pain scores of 61 mm on a VAS [2] indicating extensively pain issues. In the present study preoperative pain scores were not quite as high, but 16 out of 29 of patients reported moderate-severe pain (VAS≥30 mm) during knee flexion preoperatively (8 in the placebo group, 8 in the ropivacaine group). Preoperative pain and opioid usage are predictors for increased acute postoperative pain and opioid requirements [10], [11].

As the procedure becomes increasingly complicated with each subsequent revision and prior surgery is a predictor for increased pain [11], the demands for optimal postoperative pain treatment accumulate accordingly. Early rehabilitation and avoiding postoperative falls are important aspects of postoperative care in this population. Both of these can be facilitated by the ACB. Our institution is a tertiary referral center which carries out highly specialized functions for all parts of Denmark, and we consider it a strength that all procedures were performed by the same surgeon (HMS). However, half of the patients were 2^nd^, 3^rd^ or 4^th^ time revisions. Therefore, our study population might not be comparable with the more commonly performed 1^st^ time revisions, and this may reduce generalizability of the study.

To counter the problem of tolerance in patients treated with opioids, we decided that patients with a daily intake of strong opioids should continue with their habitual analgesics during the study period, and that these doses should not be included in the cumulative morphine consumption. By chance, more patients in the ropivacaine group (5/15 (36%) versus 2/16 (12.5%) had a daily intake of strong opioids (Table 1). Including the habitual analgesics from these patients in the cumulative total morphine consumption did not alter the results (P = 0.43). Patients treated with weak opioids may of course develop tolerance too, and as more patients in the placebo group had a daily intake of weak opioids or a non-daily intake of strong opioids, this might have affected our results. However, as there are no differences in morphine consumption between the groups, this would not have affected the conclusion of the study.

As the ACB is a novel technique, we believed it was important to compare the block with a placebo control. If we had compared two active treatments, and had found no significant difference, we would have risked concluding equal efficiency, while on the contrary neither might have been effective [12]. Although the group receiving saline is named “placebo group”, the placebo part only refers to the block procedure. Both groups received a standard basic analgesic regimen as recommended by the PROSPECT Working Group [13]. Although the FNB is also recommended by the PROSPECT Working Group, this block is rarely used in Denmark due to the concurrent motor impairment.

Previous reports of the ACB state that intermittent boluses are preferable, in order to ensure spread throughout the aponeurotic canal [5], [14]. However, intermittent boluses might be challenging in every day practice. In the current study, ropivacaine was administered as an initial bolus of 30 ml 0.75% ropivacaine, followed by an additional bolus of 15 ml 0.75% ropivacaine 6 hours later. Because we were not able to ensure proper administration of intermittent boluses thereafter, this was followed by an infusion of 0.2% ropivacaine 8 ml/h. Based upon the results in the current study it is not possible to interpret whether data are a reflection of a single injection block or continuous infusion. The ACB is a relatively new block and further research studies, including dose-ranging studies are required to find the optimal administration of local anesthetic.

Two previous studies reported a success rate of 95 and 98% for the ACB, assessed by temperature discrimination in the saphenous innervation area [5]–[7]. In contrast, a third study performed an ultrasound control scan in a subgroup of patients, on the second postoperative day, revealing catheter displacement to outside the adductor canal in 5 out of 8 patients [8]. Unfortunately, the authors did not assess for saphenous sensory block in relation to the control scan. In the current study, we did not assess for block failures, but two patients were excluded due to accidental catheter withdrawal.

## Conclusions

Pain scores were consistently lower in the ropivacaine group including clinically relevant differences between groups. However, the mean differences varied and the only statistically significant difference between our groups was seen for pain during knee flexion at 4 hours postoperatively (primary endpoint). Due to a larger than anticipated dropout rate and considerate heterogeneity in the study population (large variability in preoperative pain scores, daily morphine consumption and order of revision being performed), more studies are needed before any recommendations regarding ACB for pain relief after revision TKA can be made. Future studies should include a large enough sample size to balance the distribution of confounders, or alternatively minimize heterogeneity by in- and exclusion criteria.

## Supporting Information

Checklist S1 **Supporting CONSORT checklist (Consolidated Standards of Reporting Trials).**
(DOC)

Protocol S1 **The protocol for this trial is available in the supplementary information file.**
(DOC)

Data S1 **All data used for analysis are available in the supplementary information file.**
(XLSX)
