# Supplementary material for: Adductor Canal Block for Postoperative Pain Treatment after Revision Knee Arthroplasty: A Blinded, Randomized, Placebo-Controlled Study
Source: PLoS One. 2014 Nov 11;9(11):e111951. doi: 10.1371/journal.pone.0111951 (PMC4227669; doi:10.1371/journal.pone.0111951)
Supplement: Protocol S1 — The protocol for this trial is available in the supplementary information file. (DOC) [file pone.0111951.s002.doc]

**PROTOCOL**

**“Efficacy of Adductor Canal Block on pain and morphine consumption after revision knee arthroplasty”**

**Primary investigator:**

**Name: Pia Jæger**

**Title: MD**

**Subinvestigators:**

**Name: Zbigniew J. K. Nielsen**

**Title: MD**

**Name: Henrik Schrøder**

**Title: MD**

**Name: Jørgen Lund**

**Title: MD**

**Name: Morten Jenstrup**

**Title: MD**

**Name: Ole Mathiesen**

**Title: MD**

**Sponsor:**

**Name: Jørgen B. Dahl**

**Title: Professor**

**Name and addresses:**

- **Sponsor**:

Jørgen B. Dahl

Professor, MD, DMSc

Departmen of Anaesthesia, HOC

Rigshospitalet

Blegdamsvej 9

2100 København Ø

Telephone: 0045 3545 3475

E-mail: jbdahl@dadlnet.dk

- **Primary investigator:**

Pia Jæger

MD

Departmen of Anaesthesia, HOC

Rigshospitalet

Blegdamsvej 9

2100 København Ø

Telephone: 0045 3545 3478

E-mail: pia.jaeger@rh.regionh.dk

- **Monitor:**

Copenhagen University Hospital, GCP unit

Bispebjerg Hospital, Bygning 51, 3.sal

Bispebjerg Bakke 23

2400 København NV

Telephone: 0045 3531 3890

Fax: 0045 3531 3889

- **Pharmacy:**

Region Hovedstadens Apotek
Marielundvej 25
2730 Herlev

**“Efficacy of Adductor Canal Block on pain and morphine consumption after revision knee arthroplasty”**

**Date**

The undersigned sponsor hereby confirms that it will at all times abide by the wording of the protocol and work in accordance with the applicable rules for Good Clinical Practice.

**Name of sponsor: Jørgen B Dahl**

Professor, MD, DMSc,

Department of Anaesthesia, HOC

Rigshospitalet

Blegdamsvej 9

2100 København Ø

**Date:___________________________**

The undersigned primary investigator hereby confirms that it will at all times abide by the wording of the protocol and work in accordance with the applicable rules for Good Clinical Practice.

##### **Name of primary investigator:** Pia Jæger

MD

##### Department of Anaesthesia, HOC

Rigshospitalet

##### Blegdamsvej 9

##### 2100 København Ø

**Date:___________________________**

**INTRODUCTION AND RATIONALE** [6](#__RefHeading___Toc257450437)

**Background** [6](#__RefHeading___Toc257450438)

**Bibliography** [7](#__RefHeading___Toc257450439)

**Aim** [7](#__RefHeading___Toc257450440)

**Endpoints** [7](#__RefHeading___Toc257450441)

**ETHICAL CONSIDERATIONS** [7](#__RefHeading___Toc257450442)

**Considerations in connection with the study as a whole** [7](#__RefHeading___Toc257450443)

**Information about and consent from patients** [8](#__RefHeading___Toc257450444)

**Protection of patient data** [9](#__RefHeading___Toc257450445)

**FRAMEWORK FOR THE STUDY** [10](#__RefHeading___Toc257450446)

**Schedule** [10](#__RefHeading___Toc257450447)

**Location of the study:** [10](#__RefHeading___Toc257450448)

**Study Design** [10](#__RefHeading___Toc257450449)

**STUDY SELECTION** [10](#__RefHeading___Toc257450450)

**Inclusion criteria.** [10](#__RefHeading___Toc257450451)

**Exclusion criteria.** [11](#__RefHeading___Toc257450452)

**Financial situation** [11](#__RefHeading___Toc257450453)

**Patients' completion of and withdrawal from the trial** [11](#__RefHeading___Toc257450454)

**Reasons for patients' withdrawal from the trial** [12](#__RefHeading___Toc257450455)

**Procedure for patients who withdraw from the trial** [12](#__RefHeading___Toc257450456)

**METHODOLOGY** [12](#__RefHeading___Toc257450457)

**General treatment plan and medication dosage** [12](#__RefHeading___Toc257450458)

**Clinical assessments** [13](#__RefHeading___Toc257450459)

**MEDICATION AND MEDICATION ADMINISTRATION** [14](#__RefHeading___Toc257450460)

**Study medication** [14](#__RefHeading___Toc257450461)

**Blinding procedure, packaging and labeling** [14](#__RefHeading___Toc257450462)

**Blinding procedure, packaging and labeling** [14](#__RefHeading___Toc257450463)

**Other treatment** [15](#__RefHeading___Toc257450464)

**Procedures in case of emergencies** [15](#__RefHeading___Toc257450465)

**Medication accounts** [15](#__RefHeading___Toc257450466)

**SIDE EFFECTS** [15](#__RefHeading___Toc257450467)

**Side effects/adverse events (AEs)** [15](#__RefHeading___Toc257450468)

**Reporting of side effects of AEs and SAEs** [16](#__RefHeading___Toc257450469)

**STATISTICAL ANALYSES** [17](#__RefHeading___Toc257450470)

**Calculation of the number of patients** [17](#__RefHeading___Toc257450471)

**Data processing** [18](#__RefHeading___Toc257450472)

**DATA REGISTRATION AND RULES FOR THE CONTROL OF INVESTIGATION PROCEDURES** [18](#__RefHeading___Toc257450473)

**Case Report Forms** [18](#__RefHeading___Toc257450474)

**Monitoring** [18](#__RefHeading___Toc257450475)

**Training** [19](#__RefHeading___Toc257450476)

**ADDITIONAL REQUIREMENTS AND GENERAL INFORMATION** [19](#__RefHeading___Toc257450477)

**Insurance** [19](#__RefHeading___Toc257450478)

# **INTRODUCTION AND RATIONALE**

## **Background**

In this study we wish to investigate the effect of Adductor Canal Block on pain and morphine consumption in patients after revision knee arthroplasty.

Total knee arthroplasty is associated with intense postoperative pain and revision arthroplasty is assumed to be even more painful. The patient population consists primarily of elderly people with high co-morbidity. It is therefore essential that, in addition to effective pain relief and minimal side effects, the pain treatment is aimed at promoting functional rehabilitation and reducing postoperative morbidity. There exists a large number of treatment options for postoperative pain treatment after knee surgery (1). Everything from systemic opioids, NSAIDs, paracetamol and gabapentinoids, to central (epidural) and peripheral nerve blocks and local infiltration analgesia (2) is employed. No single approach has been shown to be generally effective and there is no "gold standard" (1).

The sensory innervation of the knee arises from n. femoralis, n. ishiadicus and n. obturatorius. N. saphenus is is the most important cutaneous branch of n. femoralis. It follows a. femoralis down into the adductor canal and passes through this to innervate the lower medial and anterior aspect of the knee and lower leg. A dissection study by Horner et al. has shown that the ramus anterior n. obtoratorius perforates m. adductor magnus and branches into the adductor canal (3). The branches supply the medial and anterior part of the femur, and to a large extent the perigeniculate structures.

Block of n. femoralis as well as plexis lumbalis has been shown to be effective for pain relief after knee surgery (1). The development of ultrasound-guided (ultrasonographic, US) blocks makes it possible to visualize the nerve and the spread of the local anaesthetic and thus potentially increase the success rate of peripheral nerve blocks (4).

As n. saphenus is the cutaneous main branch from n. femoralis, it is estimated that a block of this will be a good analgesic supplement to postoperative pain treatment after knee surgery. A simultaneous block of ramus anterior n. obturatorius will enhance the analgesic effect.

A number of different methods for block of n. saphenus have been described. These methods include studies with or without nerve stimulation as well as US-guided blocks (5-7). We want to investigate the effect of Adductor Canal Block by US-guided catheterization in the adductor canal at the mid-thigh level, with subsequent injection of Ropivacaine 7.5 mg/ml. The postoperative analgesic efficacy of such a block for total knee arthroplasty has not previously been described in the literature.

Adductor channel blocks are almost exclusively sensory blocks, since the ramus muscularis n. femoralis to m. vastus medialis is the only motor branch passing through the adductor canal. It is our expectation that this primarily sensory block will promote quick mobilization and reduce the risk of complications due to immobilization.

## **Bibliography**

1. Fischer HB, Simanski CJ, Sharp C, Bonnet F, Camu F, Neugebauer EA, Rawal N, Joshi GP, Schug SA, Kehlet H; PROSPECT Working Group. A procedure-specific systematic review and consensus recommendations for postoperative analgesia following total knee arthroplasty. Anaesthesia. 2008;63:1105-23.
2. Dahl JB, Møiniche S. Relief of postoperative pain by local anaesthetic infiltration: efficacy for major abdominal and orthopedic surgery. Pain 2009:143:7-11.
3. Horner G, Dellon AL. Innervation of the human knee joint and implications for surgery. Clin Orthop Relat Res 1994;301:221-6.
4. Tran de QH, Muñoz L, Russo G, Finlayson RJ. Ultrasonography and stimulating perineural catheters for nerve blocks: a review of the evidence. Can J Anaesth 2008;55:447-57.
5. Benzon HT, Sharma S, Calimaran A. Comparison of the different approaches to saphenous nerve block. Anesthesiology. 2005;102:633-8.
6. Krombach J, Gray AT: Sonography for saphenous nerve block near the adductor canal. Reg Anesth Pain Med 2007; 32: 369-70
7. Lundblad M, Kapral S, Marhofer P et al. Ultrasound-guided infrapatellar nerve block in human volunteers: description of a novel technique. Br J Anaesth 2006;97: 710-714.
8. Wang H, Boctor B, Verner J. The effect of single-injection femoral nerve block on rehabilitation and length of hospital stay after total knee replacement. Regional anesthesia and Pain Medicine 2002; 27:139-44.

## **Aim**

Our aim is to investigate the effect of Adductor Canal Block as a post-operative analgesic treatment after revision knee arthroplasty. Our hypothesis is that Adductor Canal Block can reduce the postoperative use of morphine, as well as reduce pain scores, side effects and facilitate mobilization, when compared with placebo treatment.

## **Endpoints**

#### Primary end points

- Pain score with 45 degree active flexion of the knee (VAS) 4 hours postoperatively.

###### **Secondary end points**

- Pain score with 45 degree active flexion of the knee (VAS) 1, 2, 4, 6 and 8 hours postoperatively, given as "area under curve" (AUC) calculation for the period 1-8 hours postoperatively.
- Pain score with 45 degree active flexion of the knee (VAS) 24 hours postoperatively.
- Pain score at rest (VAS) 1, 2, 4, 6 and 8 hours postoperatively, indicated as "area under curve" (AUC) calculation for the period 1-8 hours postoperatively.
- Pain score at rest (VAS) 24 hours postoperatively.
- Total need for morphine in the periods 0-8, 8-24 and 0-24 hours postoperatively, administered as patient controlled pain treatment (bolus 2.5 mg, lockout 10 minutes).
- Degree of nausea 1, 2, 4, 6 and 8 hours postoperatively, calculated as an average value for the period 1-8 hours postoperatively.
- Degree of nausea 24 hours postoperatively.
- Number of vomiting events during the periods 0-8, 8-24 and 0-24 hours postoperatively.
- Consumption of Zofran in the periods 0-8, 8-24 and 0-24 hours postoperatively.
- Degree of sedation 1, 2, 4, 6 and 8 hours postoperatively, calculated as an average value for the period 1-8 hours postoperatively.
- Degree of sedation 24 hours postoperatively.

# **ETHICAL CONSIDERATIONS**

## **Considerations in connection with the study as a whole**

Ropivacaine is a known and registered medicine.

The applied block is performed under ultrasound guidance and is not deemed to be associated with complications.

Patients are prescribed supplemental analgesics corresponding to current practice, and therefore there are not deemed to be ethical problems in this study.

The study will be conducted in accordance with the principles of the Declaration of Helsinki. The protocol will be submitted to the local Research Ethics Committees, the Danish Medicines Agency and the Danish Data Protection Agency for approval. The investigator will also inform the Research Ethics Committee, the Danish Medicines Agency and the Danish Data Protection Agency of any significant or major changes in the protocol. The study will be reported in the international database clinicaltrials.gov.

All patients taking part in the study must provide their oral and written informed consent before they are included in the study. It is the investigator's duty to inform patients so that they are fully aware of all aspects of the survey.

Patients may at any time withdraw their consent to participation in the survey. If the patient decides to do this, this will not impair the patient's relationship with the investigator and the patient will continue to receive the best treatment the unit can offer.

Taking part in the trial gives patients the opportunity to administer the analgesic treatment themselves, which has been shown in a large study to give a high level of patient satisfaction. It is our expectation that the Ropivacaine group (Group A) will have more optimal pain management, with fewer opioid-related side effects. Apart from the possibility of adjusting their pain treatment themselves, patients will not benefit directly from the treatment. However, it is hoped that the information gathered will result in improved pain management after revision knee arthroplasty in the future.

**Risks, side effects, disadvantages, etc.**

We believe that this study does not involve side effects or risks that exceed those normal for this operation. The drugs used in the trial are well known and have few side effects, so it is judged that participation in the trial is associated with very low risk. In the same way, we hope to reduce the need for morphine after surgery, which could also mean fewer side effects. In addition, patients receive additional analgesic treatment according to usual practice, which is why no ethical problems are seen in this study.

The applied block is inserted under ultrasound guidance and not deemed to be associated with complications.

Participation in the trial is not expected to result in additional hospitalization time or investigations than described above.

## **Information about and consent from patients**

All patients registered for revision knee arthroplasty under general anaesthesia at the Rigshospital's Department of Orthopaedic Surgery Department U will be invited to take part in the trial. The first contact with the patient will take place upon admission the day before surgery. At this point, the written information (Annex 1) is given to the patient and the patient is informed verbally about the trial. At the beginning of the meeting, the patient will be informed that they have the possibility of having a companion present. If necessary, there will be a wait until the arrival of the companion before the information interview is held. The verbal information will be given by one of the investigators, Consultant Henrik Schrøder, Consultant Zbigniew J. K. Nielsen or Doctor Pia Jæger. The interview will be conducted in a closed room without distractions or interruptions. The patient will be given time to consider the request.

Consent shall be obtained on the day of surgery at the latest. From the time the written and oral information is given, the patient will have around a day of reflection before consent is obtained.

After agreement, the trial subjects will receive comprehensive verbal and written information about the aims, processes, potential benefits and risks of the trial, as well as the block type and local anaesthetic employed, including any possible side effects that these might have. Subjects will be informed that they are entitled to a period of deliberation before consent is given, and that they have the right to have a companion present when the information is given. The subjects will also be informed that participation is voluntary and that they may withdraw from the trial at any time. Subjects will be informed as to what treatment they will receive if they choose not to participate or wish to withdraw from the trial.

Patients will be informed that randomization will take place on the day of surgery.

Informed written consent will be received from all subjects before inclusion in the trial.

A copy of the information and consent declaration will be given to the subjects.

Relevant provisions of the Research Ethics Committee regarding informed consent will be followed. The information and consent declaration for patients is attached (Annex 1).

## **Protection of patient data**

All information will be treated confidentially and in the reporting of study results, patients will be anonymous and the persons responsible for this trial are bound to confidentiality.

The investigator will create an identification list of all patients who have been given trial numbers. This list will contain the full name and CPR number (Annex 3).

Data collected in the form of a Case Report Form and records will only be made available to third parties in accordance with Danish law, which means in connection with monitoring by Copenhagen University Hospital's GCP unit, as well as upon inspection by authorized representatives of the relevant authorities.

Patients will be informed in writing that the results will be stored and analysed in a computer, that all information will be treated confidentially and that patients will be anonymous in the reporting of the results of the trial. They will also be informed that local data legislation will be observed.

Patients will also be informed in writing of the possibility of a review by public authorities in the event that they require access to relevant information and that Copenhagen University Hospital's GCP Unit will also have access to this information.

The investigator will allow the project to follow the rules of Good Clinical Practice.

# **FRAMEWORK FOR THE STUDY**

## **Schedule**

The study is scheduled to start: 01.08.10. The study is scheduled for completion: 01.02.14

## **Location of the study:**

Department of Anaesthesia and Department of Orthopaedic Surgery, Centre of Head and Orthopaedics, Rigshospitalet.

## **Study Design**

**Type of trial:** Placebo-controlled

**Randomization:** Computer-generated randomized lists, sealed envelopes, block randomization of 10 patients.

**Blinding:** Double-blind

**Participants:** Patients set to undergo knee arthroplasty under general anaesthesia

**Number:** 34 (evaluable patients)

# **STUDY SELECTION**

## **Inclusion criteria.**

Patients must meet all the following criteria to be suitable for inclusion in the study:

- Age > 40 < 85 years old
- Patients scheduled for total knee arthroplasty in general anaesthesia with Propofol and Ultiva.
- Patients who gave their written informed consent to participating in the study after having fully understood the contents of the protocol and restrictions.
- ASA 1-3.
- BMI > 18 and < 40

## **Exclusion criteria.**

Patients who meet one or more of the following criteria are not suitable for inclusion in this study:

- Patients who cannot cooperate with the study.
- Patients who cannot understand or speak Danish.
- Patients with allergy to the medicines used in the study.
- Patients suffering from alcohol and/or drug abuse – based on the investigator's opinion.

## **Financial situation**

This study was investigator-initiated. Costs in connection with the study relate to the various notifications, and the study medication. The costs of the various notifications are met by Professor Jørgen B. Dahl's research account, and the costs of study medication are met by the Department of Anaesthsia, HOC, Rigshospitalet. There will be no external support for the study. The investigator has no financial interest in the trial.

## **Patients' completion of and withdrawal from the trial**

- A patient who has completed the study is a patient who has followed the trial's treatment plan for the prescribed initial 24 hours postoperatively.
- A patient who has not completed the study is a patient included in the trial, i.e. who has given informed consent, but does not complete the trial, whether the patient has received the trial medication or not.
- If a patient does not complete the trial an account should be given as to whether and how this subject is followed in the trial – this also applies to drop-outs – as well as what data has been collected from these subjects.

## **Reasons for patients' withdrawal from the trial**

A patient can be withdrawn from the trial under the following conditions:

- If the investigator believes that a change of treatment will be best for the patient
- If the patient wishes to withdraw from the trial
- Violation of protocol rules

## **Procedure for patients who withdraw from the trial**

In accordance with the Declaration of Helsinki, patients have the right to withdraw from the trial at any time for any reason. The investigator also has the right to withdraw a patient from the trial at any time.

The reason why a patient is withdrawn from the trial before the scheduled time shall be recorded in the patient's Case Report Form.

# **METHODOLOGY**

## **General treatment plan and medication dosage**

- An examination is made of whether the patient meets the inclusion and exclusion criteria.
- The trial period is 24 hours with T0 = last suture.
- **Standard pre-medication:**
  - 1 hour before arrival in the operating theatre: Paracetamol 1 g per os.
- **Ultrasound-guided insertion of catheter in the Adductor Canal:**
  - The catheter is inserted under ultrasound guidance under general anesthesia.
- **Treatment A**:
  - Adductor Canal Block via catheter inserted with ultrasound guide, with Ropivacaine 0.75%, bolus 30 ml 0 hours postoperatively, followed by bolus with Ropivacaine 0.75%, 15 ml 6 hours postoperatively. At 6 hours postoperatively an infusion catheter is connected with an infusion of Ropivacaine 0.2%, 9 ml per hour. The pump remains connected until the end of the trial 24 hours postoperatively.
- **Treatment B**:
  - Adductor Canal Block via catheter inserted with ultrasound guide, with placebo, isotonic NaCl, 30 ml bolus 0 hours postoperatively, followed by bolus of NaCl, 15 ml, 6 hours postoperatively. At 6 hours post-operatively an infusion catheter is connected with an infusion of isotonic NaCl, 9 ml per hour. The pump remains connected until the end of the trial 24 hours postoperatively.
- **Standard anaesthesia:**
  - General anaesthesia with Propofol and Remifentanil
  - Rocuron 0.5 mg/kg for intubation, no reversal with neostigmine
  - With a fall in blood pressure, ephedrine is given, alternatively metaoxedrin
  - Fluids as needed
  - 30 minutes before the end of anaesthesia, morphine 0.2 mg/kg is given iv
- **Standard postoperative nausea and pain treatment:**
  - Given postoperatively and for the rest of the 24-hour trials starting T0 + 2 hours:
    - Paracetamol 1 g every 6 hours po.
    - Ibuprofen 400 mg every 6 hour if there are no contraindications.
  - Patient-controlled iv morphine (PCA), bolus 2.0 mg, lockout time 15 minutes. Mixture: Morphine 1 mg/ml.
  - In the first hour postoperatively upon declaration of pronounced pain, fentanyl 0.1 mg may be given iv. The dose may be repeated after 10 minutes.
  - If there is a need for additional morphine in addition to the PCA pump, in the first 2 hours postoperatively, bolus doses of 2.5 mg morphine iv can be given on request.
  - Patients who are in regular opioid therapy preoperatively continue this treatment unchanged during the period of the trial.
  - Zofran 4 mg iv at the first indication of moderate-pronounced nausea may then be supplemented with 1 mg iv.

## **Clinical assessments**

- **Patient-controlled morphine consumption**:
  - Number of bolus doses delivered in the period 0-8 hours postoperatively is recorded
  - Number of bolus doses delivered in the period 0-24 hours postoperatively is recorded
- **Pain:**
  - Patients' pain is recorded on a Visual Analogue Scale (VAS) of 100 mm, where 0 = no pain and 100 = worst possible pain. Patients state their own pain intensity.
  - Pain is recorded at rest and in connection with 45 degrees active flexion of the knee.
  - Pain is recorded preoperatively before administration of medication, as well as 1, 2, 4, 6, 8 and 24 hours postoperatively, corresponding to the end of the trial period.
- **Side effects:**
  - Nausea and sedation are recorded on a verbal scale (none, mild, moderate, severe) 1, 2, 4, 6, 8 and 24 hours post-operatively.
  - The number of productive vomiting events (volume estimated over 10 ml) is recorded corresponding to the periods 0-1, 1-2, 2-4, 4-6, 6-8 and 8-24 hours post-operatively by interview with the patient.
  - The patient's use of Zofran (mg) 0-8 and 0-24 hours postoperatively is recorded.

The study extends for each patient from introductory conversation to 24 hours postoperatively. The patient receives the above treatments in accordance with a computer-generated randomized list.

# **MEDICATION AND MEDICATION ADMINISTRATION**

## **Study medication**

Active preparation: **Ropivacaine 0.75%.**

Dispensed in the form of injection solution for perineural and epidural use. Contains: ropivacaine hydrochloride monohydrate. In addition, sodium chloride, hydrochloric acid and sodium hydroxide. Water for injection.

The preparation is imported to Denmark by: AstraZeneca A/S, Roskildevej 22, 2620 Albertslund

Holder of marketing authorisation: AstraZeneca A/S, marketing authorisation number 17973.

Active preparation: **Ropivacaine 0.2%.**

Dispensed in the form of infusion solution for perineural and epidural use. Contains: ropivacaine hydrochloride monohydrate. In addition, sodium chloride, hydrochloric acid and sodium hydroxide. Water for injection.

The preparation is imported to Denmark by: AstraZeneca A/S, Roskildevej 22, 2620 Albertslund

Holder of marketing authorisation: AstraZeneca A/S, marketing authorisation number 17971.

Placebo: Sodium chloride 9 mg/ml. Infusion liquid, infusion. 1 litre contains 9 g of sodium chloride in sterile water. Electrolyte/l: 154 mmol chloride and 154 mmol sodium. Isotonic. Osmolarity of approx. 308 mmol/l

## **Blinding procedure, packaging and labeling**

## **Blinding procedure, packaging and labeling**

The study is performed as a double-blind randomized study. Randomisation will be performed by the Capital Region Pharmacy. The experimental medicine will be packed and labelled by the Capital Region Pharmacy. in accordance with the rules for this. Each patient receives medication as indicated below under **"General treatment plan and medication dosage"**. Ropivacaine 0.75% or placebo are blinded by being packaged in one blinded closed cardboard box. The cardboard box is handed over to a person not involved in the trial, who records the batch number and expiry date. This person then draws the medicine into a neutral spray and returns it to the patient's anaesthesia personnel for administration. 2 sets of additional sealed code envelopes are prepared. These contain information about which treatment each of the subjects is being randomly assigned. One set is retained by the sponsor, while the other is retained by the investigator at the Department of Anaesthesia, HOC, Rigshospitalet, in a secure locked place. Capital Region Pharmacy retains the non-blinded randomization list, which will only be sent to the investigator when the data has been analysed.

## **Other treatment**

Other medications that are considered necessary for the subject will be given at the discretion of the investigator.

Administration of all other medical treatment will be recorded on the accompanying Case Report Form.

## **Procedures in case of emergencies**

The investigator will ensure that there are the necessary procedures and expertise to tackle an emergency situation that may arise during the study.

The blinding may only be broken if the continued treatment of the patient requires knowledge of the randomization code. If the code envelope is opened, the date and reason shall be recorded and the envelope signed by the investigator. This results in the exclusion of the patient.

## **Medication accounts**

The investigator will ensure that the study medication is kept in a safe place and only provided to participants in this study. The investigator will ensure that there is strict accounting of the study medication supplied. The investigator shall give an account of any medication that accidentally or otherwise goes missing, and for any discrepancy between the supplied and returned medications.

# **SIDE EFFECTS**

## **Side effects/adverse events (AEs)**

Side effects are defined as any adverse event, signs or symptoms that occur during participation in the study which are time related to the administration of the study medication, whether the adverse event is considered to be related to the study medication or not. All adverse events must be recorded in the patient's Case Report Forms. If an adverse event occurs more than 24 hours after the last administration of the study medication, and there is no apparent causal connection or relation to the study medication, this shall not be deemed to be an adverse event.

Start and end date/time, severity and effects after the study medication must be recorded for all incidents. The severity of the adverse event and the relationship with the study medication must be assessed in accordance with the guidelines described below.

The investigator must assess the relationship between an adverse event and the study medication using the following guidelines:

**Guidelines for adverse events' possible relationship to treatment:**

1. Not related – no temporal relationship, other etiologies are very likely the cause
2. Possibly related – less clear correlation, other etiologies are also possible
3. Probably related – clear temporal correlation with improvement on discontinuation of medication, and not reasonably explained by the patient's known clinical condition.
4. Related – clear temporal relationship with repeated treatment test or clinical assessment.

Patients with adverse events will be monitored with appropriate clinical assessments and laboratory tests according to the decision of the attending doctor. All adverse events will be followed until satisfactory recovery or stabilization.

A serious adverse event (SAE) is understood to be any incident involving a significant risk of the death or disability of the patient (or their offspring), including, but not limited to, an event that results in:

- death
- is life-threatening – in the investigator's opinion the patient was in immediate risk of death from the adverse event when it appeared
- requires hospitalization or prolongs existing hospitalization
- is permanently disabling

- is a congenital anomaly

A summary of product characteristics for Naropin from the Danish Medicines Agency's website will be used as a reference document when it is assessed whether a serious related adverse event is expected or unexpected.

**Grading of adverse events:**

The medical investigator should attempt to identify all clinical and objective reactions from patients in the treatment and determine their relationship with the study medication. Reactions, if there are any, should be graded according to the following scale:

1 = slight

2 = moderate

3 = severe

4 = life-threatening

## **Reporting of side effects of AEs and SAEs**

The investigator is responsible for ensuring that all adverse events are recorded in the patient's Case Report Form.

The sponsor is responsible for ongoing monitoring of the study's risk/benefit relation. If there arise or are seen to be situations that may affect the safety of the study subjects or the performance of the study, these must be **always** immediately reported to the Danish Medicines Agency. Similarly, reports should also be made to all investigators and Research Ethics Committees involved.

In addition, the following rules apply for reporting to the authorities. Events and reactions should be included for all study medication – including the experimental treatment, the comparative treatment and any placebo.

The sponsor shall at all time be kept informed by the investigator of any adverse events. At the end of the trial, the final report shall contain a description of all side effects.

Serious adverse events – SAEs - should be reported immediately by the investigator to the sponsor (sponsor-investigator). SAEs must be reported annually by the Investigator to the Research Ethics Committee throughout the whole trial period, together with a report on the safety of the trial subjects.

Serious adverse reactions – SAR (serious presumed side effects) - shall be reported by the sponsor (sponsor-investigator) annually to the Danish Medicines Agency. The report shall also include a report on the safety of trial subjects.

Suspected unexpected serious adverse reaction – SUSAR (Unexpected and serious side effects) - must be **immediately** reported by the sponsor (sponsor-investigator**)** to the Danish Medicines Agency.

Fatal or life-threatening SUSARs must be reported within **7 days** of the sponsor becoming aware of them, and within **8 days** of the report the sponsor must inform the Danish Medicines Agency of all relevant information on the follow-up.

All other unexpected serious suspected adverse reactions should be reported to the same authorities within 15 days of the sponsor (sponsor-investigator) being informed of these.

All reports must be accompanied by comments on any consequences there are for the trial.

It is also recommended that the sponsor (sponsor-investigator) should inform the manufacturers of the medication.

# **STATISTICAL ANALYSES**

## **Calculation of the number of patients**

Results from previous studies suggest that all patients will have pain postoperatively. Average pain scores (VAS) for activity (45 degree flexion of knee) in the first 24 hours based on previous studies is typically around 75 (SD 20) (8). We assess that a reduction of 20 in the pain score (VAS) will be clinically relevant.

With a type 1 failure risk of 5% and a type 2 failure risk of 20%, a total of 34 patients, i.e. 17 in each group, will be required.

It is planned to include a total of 34 evaluable patients in the measure of main effect. To compensate for drop-outs it is aimed to include 36 patients.

## **Data processing**

The trial will be completed when 34 evaluable patients are included in the study.

The evaluability of each patient in the statistical analyses will be decided before the code is broken. Only subjects for whom data for pain scores at 45 degrees flexion of the knee 0-8 hours postoperatively is available will be included in the statistical analysis. Excluded patients and missing, unused or false data will be described. Data will be stored and evaluation and statistical analysis will be made by computer, where patient anonymity will be preserved and local data legislation will be observed.

Results will be described with mean values and standard deviation of the mean value, as well as median and inter-quartile values. A 5% level of significance will be used. Binomial data will be analysed with Fisher's exact test and ratio-interval data with the Mann-Whitney test. Multiple analyses will be adjusted with the Bonferroni test. Pain scores will be compared with the AUC calculation. Any change to the statistical plan will be accounted for by publication.

# **DATA REGISTRATION AND RULES FOR THE CONTROL OF INVESTIGATION PROCEDURES**

The study will be conducted in accordance with the applicable rules on clinical trials involving people in respect of quality control and quality management, and will follow the Good Clinical Practice guidelines.

The investigator and co-investigators at the hospital are responsible for managing and archiving data in accordance with current regulations. The data belongs to the investigator and co-investigators at Rigshospital, Anesthesia and Surgical Clinic 4231, HOC.

## **Case Report Forms**

A Case Report Form (CRF) will be completed for each patient included in the study. This will be signed by the investigator to confirm the accuracy of the data. Corrections to the data will only be made by crossing out the incorrect data (the incorrect information will remain visible and legible) with the correct data written next to the deleted data. Correction fluid will not be used. Corrections will be dated and signed by the investigator or their deputy.

Source data is defined as: CRF, health records, medication and observation form from the intermediate unit and anesthesia record.

## **Monitoring**

Copenhagen University Hospital GCP unit

Bispebjerg Hospital, Building 51, 3rd floor

Bispebjerg Bakke 23

2400 Copenhagen NV

Tel. 3531 3890

## **Training**

The investigator will ensure that the personnel involved are appropriately trained and qualified, and have the necessary information to carry out the study.

# **ADDITIONAL REQUIREMENTS AND GENERAL INFORMATION**

# **Insurance**

For patients at Rigshospitalet, the Department of Anesthesia, HOC, Rigshospitalet, accepts statutory responsibility on behalf of the investigator and the investigator's colleagues for any injury caused directly or indirectly by the study medication in this clinical study, provided that the investigator and the investigator's colleagues have followed the instructions given in this protocol and any supplements thereto, and that the investigator and the investigator's colleagues conducted the study scientifically and in accordance with applicable rules and accepted techniques. In the event of injury or death unrelated to the conduct of the study, patients are covered by the hospital's insurance.

**Publication of results**

On the basis of the data the investigator will write a report of the study. This report will be forwarded to the relevant authorities. The report will also form the basis of a manuscript to be submitted for publication with the following order of authors:

1. **Pia Jæger**
2. **Zbigniew J. K. Nielsen**
3. **Henrik Schrøder**
4. **Jørgen Lund**
5. **Morten Jenstrup**
6. **Ole Mathiesen**
7. **Jørgen B. Dahl**

Both negative and positive results will be published.
